# Epidermal Differentiation Complex: A Review on Its Epigenetic Regulation and Potential Drug Targets

**DOI:** 10.22074/cellj.2016.3980

**Published:** 2016-04-04

**Authors:** Sinha Abhishek, Suresh Palamadai Krishnan

**Affiliations:** School of Bio-Sciences and Technology, VIT University, Vellore, Tamil Nadu, India

**Keywords:** Keratinocyte, Proliferation, Differentiation, Cornified Envelope, Drug Targeting

## Abstract

The primary feature of the mammalian skin includes the hair follicle, inter-follicular epidermis and the sebaceous glands, all of which form pilo-sebaceous units. The epidermal protective layer undergoes an ordered/programmed process of proliferation and differentiation, ultimately culminating in the formation of a cornified envelope consisting of enucleated corneocytes. These terminally differentiated cells slough off in a cyclic manner and this process is regulated via induction or repression of epidermal differentiation complex (EDC) genes. These genes, spanning 2 Mb region of human chromosome 1q21, play a crucial role in epidermal development, through various mechanisms. Each of these mechanisms employs a unique chromatin re-modelling factor or an epigenetic modifier. These factors act to regulate epidermal differentiation singly and/or in combination. Diseases like psoriasis and cancer exhibit aberrations in proliferation and differentiation through, in part, dysregulation in these epigenetic mechanisms. Knowledge of the existing mechanisms in the physiological and the aforesaid pathological contexts may not only facilitate drug development, it also can make refinements to the existing drug delivery systems.

## Introduction

The epidermal layer is the outermost functional barrier, which plays a crucial role in protecting the human body from various environmental insults (e.g. trauma, infection, physical and/or chemical injury including exposure to excessive radiation and temperature) by undergoing a series of terminal differentiation-steps leading to programmed cell death. The cells in the inter-follicular region (stratified squamous epidermal keratinocyte cells) undergo a set of intrinsically programmed proliferation/differentiation events (genomic, proteomic and structural). These events are initiated by the mitotically active stem cells (with self-replicating and differentiating potential) in the basal layer subsequent to receiving appropriate physical and biochemical cues. During this ordered sequential process, the cells detach from the basal lamina and form part of the suprabasal layers (spinous, granular and the stratum corneum). The stratum corneum, with the terminally differentiated dead enucleated corneocytes, encases and protects the human body as a shield. This protective cover is like "a Saran Wrap" guarding against the entry of foreign agents, apart from preserving the moisture content within. Their final destination is the tissue surface where these dead cells finally get sloughed off from the surface (a cyclic phenomenon for about 4 weeks). This complete journey of homeostasis-cum-replenishment of the cells depends critically on the self-renewing capability of the stem cells, which together with their differentiated progeny are arranged in columns [epidermal functional units (EFU)]. Following injury, these epidermal stem cells as well those in the bulge region of the hair follicle are known to participate in tissue repair processes. However, only the stem cells in the bulge region are multipotent (i.e. capable of differentiating into all the three aforesaid differentiated progenies) (1). However, deregulation of this sequential process can yield to a dysfunctional barrier, leading to skin disorders such as psoriasis, atopic dermatitis (2) and even skin-related neoplasia (3). 

### The epidermal differentiation complex

Epigenetic mechanisms play an important role in the growth and differentiation of keratinocytes by regulating the expression of genes in a 2 Mb region of human chromosome 1q21. The cassette of genes present in this region, known as the epidermal differentiation complex (EDC), are responsible for epithelial tissue development and repair by regulating the terminal differentiation program of the keratinocytes through a series of coordinated and inter-dependent signal transduction pathways. These genes encode structural proteins, including involucrin, loricrin and small proline-rich proteins, responsible for the cornified envelope of the skin and also a number of calcium binding proteins including intermediate-filament associated profilaggrin and trichohyalin, and several S100A proteins (4,6). During terminal differentiation, the epidermal keratinocytes undertake a series of programmed cellular events which allows them to become part of the cornified layer of keratinized ´inactive´ corneocytes from the basal layer of ´active´ keratinocytes. A pool of epigenetic modulators influences this programming via induction or repression of EDC gene(s), thus playing a crucial role in epidermal development. 

### The epigenetic machinery modulating the epidermal differentiation complex expression

The epigenetic regulators exhibit their functions through various mechanisms, each employing a unique kind of chromatin re-modelling factor or an epigenetic modifier, and may act individually and/or combined (7). They have been grouped according to their mechanism of action such as ATPdependent and higher order chromatin re-modelling which result in differential gene expression in discrete compartmentalized domains. Other major mechanisms include DNA methylation and covalent histone modifications (8). 

A major class of enzymes in the category of histone regulators include histone deacetylases (HDACs, HDAC1 and HDAC2). These enzymes catalyze the removal of acetyl groups from histone proteins (histone deacetylation) leading to gene repression. This repression is due to the facilitation of histone proteins to bind DNA (with the ensuing chromatin condensation), resulting in gene silencing due to the reduced accessibility of regulatory elements to transcription factors. In contrast, histone acetyl transferases (HATs) acetylate histone proteins and facilitate unfolding of the chromatin, thus providing better access of the transcription factors to the promoters of genes. Any aberration in the functioning of these epigenetic processes (singly and/or combined) has been reported to adversely affect the proliferation and differentiation of keratinocytes, which in turn affects the phenotype of the resulting skin epithelium (9). Hence, epigenetic studies involving keratinocytes and the EDC may provide mechanistic insights, with relevance to ex vivo model development and drug testing. This aspect is underscored by the following examples cited. 

### Epigenetic regulation by chromatin remodelers

It has been reported that Brahma (BRM) or Brahma-related gene 1 (BRG1) ATPases, both involved in chromatin re-modelling, have partially overlapping functions in keratinocyte terminal differentiation. However, both genes are not involved in the early stages of this process but severely impair the final stage of terminal differentiation leading to skin barrier defects (10). Further, under this p63-regulated BRG1-dependent chromatin remodelling programme (by repositioning at a specific EDC locus), another genome organizer, SATB1, is also activated to induce transcription of genes required for the terminal differentiation of keratinocytes (11). Further, regulatory subunits of the switch/sucrose non-fermentable (SWI/ SNF) complex include the actin-like 6A protein/ Brahma-associated factor (ACTL6a/BAF53A). These proteins along with the catalytic subunits (BRG1, BRM and BAF250a) are involved in suppressing differentiation and maintaining the progenitor state in epidermal cells. Specifically, BRG1 and BRM are prevented, by ACTL6a, from binding to the promoter of KLF4 – an important gene whose upregulation induces differentiation (12). The chromatin remodeler (Mi-2β) is important for the self-renewal of epidermal precursors during early embryogenesis. However, during the later gestational stages, this protein is dispensable for repopulating epidermal stem cells (involved in the repopulation and differentiation of their daughter cells into the inter-follicular epidermis) (13). There is therefore a need to systematically and comprehensively catalog various chromatin remodelers at discrete stages in the epidermal terminal differentiation processes in both physiological and pathological states. 

### Epigenetic regulation by histone modifiers

HDAC1 and HDAC2 are known to be involved in hair follicle specification as well as epidermal development and stratification. Further, they have also exhibited p63and p53-repressive functions (14), providing indirect evidence of their involvement in possibly regulating SATB1. As stated above, this epigenetic modifier is known to regulate expression of the genes involved in terminal differentiation. A recent study, has shown that, in TERT-immortalized (N-TERT) and HaCaT cells, the deficiency of the aryl hydrocarbon receptor nuclear translocator (ARNT) down-regulates the amphiregulin/epidermal growth factor receptor pathway, at least in part, by activating HDAC1, HDAC2 and HDAC3. Corroborative evidence for the role of HDACs was obtained when a specific HDAC inhibitor (trichostatin A) was able to activate the amphiregulin/EGFR pathway, in cell-based systems by compensating for the ARNT deficiency. This pathway is involved in keratinocyte differentiation and hence has implications in related disorders (e.g., psoriasis and cancer), in which there is an imbalance between proliferation and differentiation (15). This finding has prompted the development and/or refinement of HDAC inhibitors as dermal anti-cancer drugs. It has been shown that enhancer of Zeste homologs 1 and 2 (EZH1/2) are involved in the regulation of genes (filaggrin, involucrin and loricrin; part of the EDC) in epidermal progenitors (16). This finding provides yet another line of evidence for this epigenetic modifier to be involved, at least in part, in the temporal and spatial step-wise terminal differentiation program. EZH 1/2 are believed to be repressed by chromobox homolog 4 (CBX4) ( part of the polycomb repressive complex (PRC1), while they downregulate Bmi1 as well as DNMT1. This thus indicates the involvement of EZH 1/2 in the EDC gene regulation process (17). 

### Epigenetic regulation by DNA methylation/ hyper-methylation

DNA methyl transferases (DNMTs), a category of epigenetic modifiers, are involved in the addition of methyl groups to the cytosine of double stranded DNA, and when such methylation occurs on the promoter region of a gene, it usually leads to epigenetic silencing of the gene. DNA methyltransferase 1 (DNMT1) is enriched in undifferentiated epidermal progenitor cells. Its depletion accordingly leads to these cells exiting the proliferative compartment and undergoing inappropriate differentiation (18). Conversely, demethylases work in removing methyl groups from DNA and histone proteins, thus activating their transcription. In fact, in psoriasis, differentially methylated CpG sites (mapped to EDC) were found to distinguish normal cells from psoriatic cells (19). In another study, analyzing whole genome methylation patterns in involved and uninvolved skin in psoriatic patients showed that hypermethylated regions corresponded to those genes involved in immune regulation, cell cycle and apoptosis, and the expression level of 2 genes [programmed cell death 5 (*PDCD5*) and tissue inhibitor of metalloproteinases 2 (*TIMP2*)] correlated with their methylation profile (20). Jumonji-AT rich interactive domain 2 (JARID2) was reported to contribute to PRC2-mediated inhibition of differentiation genes at H3K27 trimethylation-marked chromatin-repressive sites (neonatal epidermis). In adults, JARID2 is involved in the increased proliferation of hair follicle stem cells and their progeny (21). Calcium-induced differentiation in primary human keratinocytes was associated with erasure of the H2K27Me3 epigenetic imprint by a demethylase of the Jumonji family (JMJD3). Corroborative evidence for this was obtained from the blockade of differentiation consequent to the depletion of JMJD3 in organo-typic human skin tissue (22). Grainyhead-like 2 (GRHL2) has been reported to downregulate EDC expression by increasing methylation of histones at the target EDC gene promoters. This is in accordance with its role in enhancing replicative lifespan of the keratinocytes by regulating hTERT expression. GRHL2 expression is mostly restricted in the basal layer of normal epithelia whereas strong expression has been reported in upper layers of epithelial tissue in psoriatic skin type (23). N-lysine methyltransferase Setd8 (Histone H4 mono-methylation at lysine 20) is an anti-apoptotic epigenetic regulator that is controlled by c-MYC (a protein involved in cellular epidermal differentiation) and has been reported to be crucial for normal tissue homeostasis as part of a functional terminal differentiation program. The downstream target of Setd8 is thought to be p63 since its regulation is lost in Setd8-null cells (24). 

### MicroRNAs involved in epigenetic regulation of epidermal differentiation complex gene cluster

MicroRNAs (miRNAs) are capable of posttranscriptional gene regulation by binding to the 3´ untranslated region of their target mRNAs, leading to suppression of translation. Various miRNAs have been reported to play critical role in epidermal development and skin pathologies by regulating differentiation. For example, miR203 is involved in controlling early commitment of human embryonic stem cells to the keratinocyte lineage (25) and promoting epidermal differentiation and deregulating proliferation by rapidly inducing the cells to exit cell cycle, a hallmark of epidermal differentiation. They are also reported to repress and regulate p63 expression by binding to its 3´ UTR. This regulation is crucial for maintaining the proliferative and differentiation potential of basal keratinocytes and precursor cells (26,27). Once terminal differentiation has been initiated; miR203 expression is induced via the PKC/ AP-1 pathway (28) which leads to the suppression of p63 and thus halting the proliferative ability of the keratinocytes. Deregulation of miR203 can lead to pathological conditions as reported in psoriatic plaques where miR203 has been observed to be up-regulated. As a result, suppressor of cytokine signalling 3 (SOCS-3), which is involved in immuneand keratinocyte-related functions and also a downstream target of miR203, is shown to be downregulated, thus providing a link between miR203 and keratinocyte dysfunction in psoriasis (29). While miR203 may be an important marker for psoriasis and wound re-epithelization (30), its co-expression with other miRNA has also being reported in epidermal pathological conditions such as psoriasis, eczema and atopic dermatitis. In psoriatic skin, miR-203, miR-146a and miR-21 were found to be upregulated, but miR-125b displayed reduced expression. MiR-146a is found to modulate tumor necrosis factor-α (TNF-α) signalling in skin by inhibiting the expression of key protein of this pathway and thus perceived to play a role in the pathogenesis of psoriasis (29). Increased miR-21 expression in psoriasis was reported to be a result of diminished Jun/AP-1 transcriptional activity. Consequent to the over-expression of miR-21, epidermal tissue inhibitor of matrix metalloproteinase-3 (TIMP-3) expression was reduced and hence its physiological functions (regulation of stromal remodelling, angiogenesis and regeneration of epidermis) were affected (31). Terminal differentiation-induced non-coding RNA (TINCR), a human long ncRNA (lncRNA), is thought to control epidermal differentiation by regulating the mRNA of key differentiation genes from the EDC cluster (32). 

It is clear from the aforesaid examples that epigenetic mechanisms can deregulate expression of EDC (33), and this aberration in turn has been linked with skin disorders like psoriasis and skin cancer, both known to be linked to aberrations in proliferation and differentiation. Hence, this review further underscores the need for a thorough understanding of the epigenetic mechanisms (in model systems that can mimic *in vivo* conditions) that regulate EDC. This approach is crucial for target identification and/or validation. Such targets, by epigenetic modulation, would therefore be useful for reverting aberrantly functioning cells to normal cells or may facilitate programmed cell death by possibly mimicking physiologically normal terminal differentiation. 

### Epigenetic regulators as drug targets

Various drugs targeting these epigenetic elements are being studied and many have gone through clinical trials (34,35). HDAC-I inhibitors can play a pivotal role in psoriatic treatment as HDAC-I were found to be over-expressed in such skin types (36). Vorinostat and Romidepsin, both HDAC inhibitors, were developed for the treatment of cutaneous T cell lymphoma, by down-modulating IL-10 expression (37). Other drugs are in various phases of clinical development. Since a number of miRNAs play a role in homeostatic mechanisms as well as in dermal pathological conditions, they have also been considered as drug-targets. For instance, targeting miR-21 with locked nucleic acid (LNA)-modified anti-miR-21 compounds have reportedly shown to ameliorate the psoriatic phenotype in mouse models (31). Luteolin and quercetin, two plant derived flavonoids have been proven to be anti-cancer candidates by their ability to arrest cell cycle and induce cell death (38) through their interaction with eukaryotic topoisomerase I and II (39,40). Linn has also reported that oleanolic acid and leaf extracts of *Annona muricata* induce cell death and display anti-neoplastic activity in normal and immortalized epidermal keratinocytes (41,42). Both dermal cancers and psoriasis have aberrations in their differentiation program and apoptosis pathways, with epigenetics playing a pivotal role. Hence, the bioactivity of the aforesaid ethnic-based, naturally occurring, anti-cancer molecules and plant extracts (especially flavonoids) should be thoroughly evaluated in terms of their role in modulating key epigenetic genes involved in the reversal of these human pathological conditions (43). 

## Conclusion

Epigenetic mechanisms regulate expression of genes and their aberrant functioning is implicated in multiple disorders including tumorigenesis. With respect to epidermal development, epigenetic regulators affect functioning of genes (especially those in EDC) that are associated with proliferation and differentiation of keratinocytes as well as their progenitor cells including multipotent stem cells. This field has already been established as an important area of drug development as some of these key events are markers associated with drug response (e.g. demethylases and HDAC inhibitors). Elucidation of the comprehensive dermal epigenetic signatures would pave the way for "tailor-made medicine" and may serve to complement the ongoing efforts of target refinement. Despite several epigenetic drugs being in the clinical trial phase, the role of purified bioactive components (especially flavonoids) remains controversial. This therefore provides an impetus to evaluate such natural molecules (singly and/or combined) that may mimic more closely the relative stoichiometry of these health-promoting biomolecules in fruits and vegetables. This strategy may aid the development of novel, selective drugs/polydrug formulations which can treat skin disorders with minimal side effects apart from refining existing targets and drugs. 
